# MyCompass in a Swedish context – lessons learned from the transfer of a self-guided intervention targeting mental health problems

**DOI:** 10.1186/s12888-019-2039-1

**Published:** 2019-01-31

**Authors:** Anders Nilsson, Karolina Sörman, Josefin Klingvall, Emma Ovelius, Jonas Lundberg, Clara Hellner

**Affiliations:** 1 Centre for Psychiatry Research, Department of Clinical Neuroscience, Karolinska Institutet & Stockholm Health Care Services, Stockholm County Council, Norra Stationsgatan 69, 7tr, SE-113 64 Stockholm, Sweden; 2 Lumell Associates AB, Stockholm, Sweden

**Keywords:** Internet-based intervention, *myCompass*, Depression, Anxiety, Stress

## Abstract

**Background:**

Depression and anxiety is a major public health problem, in Sweden and internationally. Internet-based interventions are increasingly acknowledged as promising approaches for individuals with varying degrees of mental health problems. We present findings from the implementation of *myCompass*, a fully automated self-guided intervention of Australian origin, in a Swedish context.

**Methods:**

We (*i*) share our experience of the E-health study platform (i.e., regarding security aspects, functionality) to which the *myCompass* intervention was linked, and (*ii*) report findings from the empirical evaluation of *myCompass* (i.e., prerequisites, execution, study outcomes), in a community sample of individuals (*N* = 837) reporting mild-to-moderate levels of depression, anxiety and stress. Outcomes were calculated with repeated measures ANOVA and linear mixed models.

**Results:**

The E-health study platform proved to be an efficient tool enabling randomization, informed consent and evaluation to be administered in a fully automated manner. The study rendered substantial interest initially with 1207 individuals enrolling, however it failed to maintain engagement of those enrolled with only few participants logging in more than once or twice following registration. A smaller subgroup of “active users” (*n* = 35) had a markedly higher activity in the program, however their treatment results were not significantly better than those of the control group.

**Conclusion:**

Based on the large number of dropouts and also modest use of the intervention overall, only tentative speculations can be made regarding its effectiveness in a Swedish context. The number of individuals remaining active in the intervention is much more limited that the number of individuals initially signing up. Moreover, the transportation of interventions across countries and cultures may need more careful consideration, and pilot-trials before attempting large-scale trials are recommended.

**Trial registration:**

MyCompass was retrospectively registered at ClinicalTrials.gov. NCT03659630 September 3rd 2018, and was given the protocol ID 2015/1268–31/2 + 2016/88.

## Background

Mental health problems are one of the leading causes of disability internationally [1]. Globally, an estimated 17% of the adult population has been affected by at least one mental disorder during a 12 month period [2]. Specifically, depression and anxiety constitute a major public health issue, with depressive disorders alone being one of the leading causes of disability worldwide [3]. Depression and anxiety are commonly associated with a range of negative consequences in the daily life for the afflicted individual (e.g., reduced life quality, social and work related functional impairments, risk of somatic and psychiatric comorbidity). They are also associated with high societal costs in the form of health care consumption and loss of production [4]. Moreover, psychiatric diagnoses including depression and anxiety is the most common reasons for long-term sickness absence in developed countries [5].

### Internet-based interventions

While there are evidence-based psychosocial interventions for several common mental disorders (e.g. anxiety and depression) it is unlikely that traditional face-to-face interventions alone will solve the disease burden of mental disorders. Despite the availability of different types of traditional treatments for various mental health conditions, a common finding is that few people seek care [6]. This might be due to deficient knowledge regarding symptoms, lack of access to health care, fear of stigma, cost of treatment, and a will to handle problems oneself [7–10]. Also, the sheer number of individuals who suffer from mental illnesses calls for complementary approaches and prevention strategies. Internet based interventions could help overcome several of these obstacles since they are accessible, flexible, anonymous, cost-effective and not confined to a particular clinic.

Internet based interventions are heterogeneous, with respect to: (*i*) type of problem being targeted, (*ii*) how active the user is expected to be, (*iii*) technical properties of the platform used, and (*iv*) ways in which the intervention is delivered and managed [11]. Some interventions are technologically simple, consisting of texts with accompanying exercises, while others include more advanced solutions such as interactive exercises, smartphone applications, animated videos and the possibility to chat with a therapist [11–13]. Furthermore, some interventions are designed to provide tailored but basic information on one’s habits, such as the drinking feedback website www.checkyourdrinking.net [14], while others provide a more full-scale treatment including hefty text materials and exercises accompanied with extensive support via e-mail, chat and scheduled telephone calls [11].

Most internet based interventions to date are developed to target a specific condition (e.g., depression or social phobia), but there are also transdiagnostic interventions designed to focus on problems common across disorders [15]. Barak, Klein [11] proposed three broad categories of internet-based interventions: (*i*) interventions mainly aimed at education, (*ii*) self-guided interventions without a therapist, and (*iii*) therapist-guided interventions. Another categorization is based on whether the intervention could be regarded as general support or a regular health care intervention (i.e., which in most cases requires that the caregiver keeps a journal or chart documenting the status of the patient and all actions taken on behalf of the professional). The line between “advice and support” versus “health care” is not always clear-cut, and legislation may differ between countries. In Sweden, therapist-guided digital interventions are usually regarded as regular health care.

Since the implementation of the earliest internet-based intervention in the late 1990’s, the number of publications on their effectiveness with regards to mental health conditions has increased steadily [16]. Multiple studies have demonstrated that therapist-guided interventions could yield treatment results comparable to face-to face treatments for a wide variety of conditions across trials [17–21]. For example, a systematic review and meta-analysis of 13 studies on both somatic and psychiatric conditions (e.g., social anxiety disorder, panic disorder, tinnitus) demonstrated that guided internet-delivered cognitive behavior therapy (ICBT) and face-to-face CBT demonstrated similar effects (Andersson et al., 2014). A recent meta-analysis of individual change in 24 RCTs with internet-based interventions stated that the effects are not just statistically significant, but also clinically meaningful [22]. But internet-based interventions, as well as face-to-face treatments, have also been noted to create negative side effects for a small minority of participants [23]. One possible direction forward proposed by Muños et al. (2018) is to offer “digital apotecharies” where a plethora of evidence-based interventions for a wide range of problems is offered to the patient [24].

As one of several options, self-guided interventions could play an important role in preventing and treating psychological ill-health. Self-guided interventions have generally provided significant results, however not on par with face-to-face treatments [20, 25, 26]. Self-guided interventions are associated with specific advantages including the possibility to provide evidence-based interventions to an indefinite number of clients to a low cost. It could also suit participants who are reluctant to disclose details about their private life to therapists, as well as participants who value the flexibility self-guided interventions can provide. One example is *MoodGym*, targeting depression, with an estimated 1.2 million users in five countries [27]. Some patients might prefer self-guided over therapist guided interventions [28], and self-guided interventions could complement more traditional alternatives by providing self-help tools well suited for individuals with mild to moderate problems. Internet-based interventions, particularly self-guided ones, commonly suffer from high rates of attrition [29] however, even though there are exceptions [30].

### myCompass

*MyCompass* is a self-guided intervention of Australian origin, specifically designed for individuals with mild to moderate symptoms of stress, depression and anxiety (see Proudfoot, Clarke [31] and Harrison, Proudfoot [32] for detailed descriptions). This patient group could be particularly important to target, given that they represent approximately three quarters of individuals with mental health problems according to the Organisation for Economic Co-operation and Development [33]. Also, most internet-based interventions have focused on this degree of impairment (Andersson & Titov, 2014). The *myCompass* intervention is based on cognitive behavioral therapy, and encompasses aspects of problem solving therapy, interpersonal psychotherapy, and positive psychology (Proudfoot et al., 2013). In the sole empirical evaluation of the program published to date, community members (*N* = 720) were randomized into one of three fully automatized interventions: (*i*) *myCompass* (*ii*) a control intervention, and (*iii*) a wait list. Assessment of participants’ symptoms and functioning was done at baseline, post-intervention and at three months follow-up. Compared to the control groups, participants in the *myCompass* group demonstrated significant improvements with regards to symptoms of depression, anxiety, stress, and also improved work capacity and social function [31]. At the end of the intervention, participants in the *myCompass* group had mean symptomatic levels approaching levels in the general population, showing stability 90 days later [31]. This indicates that the program can contribute to significant health improvements, with decreased levels of mental ill health. A follow-up analysis concluded that *myCompass* was highly cost-effective and could help reduce demands on regular psychiatric services [34]. There was a high level of dropout in the *myCompass* group, however, and varying rates of engagement among the participants [31]. Given these preliminary positive findings, evaluations of the program in additional contexts are needed.

## Methods

### Aim

The aim of the current article is twofold: (*i*) to describe characteristic features of the E-health study platform (i.e., regarding security aspects, functionality) to which the *myCompass* intervention was linked, and (*ii*) to report findings from the empirical evaluation of *myCompass* (i.e., prerequisites, execution, study outcomes), in a community sample of individuals in Sweden reporting mild-to-moderate levels of depression, anxiety and stress. Our goal with describing both the platform and the intervention is to contribute with knowledge that may be valuable to researchers and clinicians designing and implementing internet-based interventions targeting various patient groups across different cultural contexts.

### Background

Based on the overall successful results in Australia, an initiative was taken by the Swedish Association of Local Authorities and Regions and the Swedish Ministry of Health and Welfare to evaluate novel treatment forms and targeted mental health interventions. *MyCompass* was translated into Swedish and launched in December 2015 with the aim to provide an easily available support intervention independent of the health care system for individuals with mild to moderate symptoms of stress, depression and anxiety. A research study to explore its effectiveness was designed. The Regional Ethical Review Board in Stockholm approved the study (# 2015/1268–31/2 + 2016/88).

#### The E-health study platform

The *myCompass* intervention was connected to the digital E-health study platform (ehalsostudie.se). This platform allows for a completely automated study process from enrollment to evaluation, with no assistance from the research team.

### Security aspects

The platform functions as a type of “gateway” for different types of interventions. Each study is hosted on a separate database, and no web application can reach data that does not belong to a particular study. Technically, the platform was developed using two layers: a web-based application and a database, which in turn are hosted on two separate servers. These two layers are separated by a firewall that overlooks the traffic in between layers. Another unique feature is that the E-health study platform uses a citizen identification system called Bank-ID (Eaton et al., 2017). Bank-ID is connected to the individual mobile phone number and is regarded as a way of identification that provides the same level of security as a passport or a driver’s license, with the difference that the issuing bank guarantees the individual’s identity. This enables the participant to enroll in the study and sign the informed consent electronically, with their identity being authenticated by a third party.

### Platform functionality

The platform provides a basis for time-efficient studies through offering functions for fully automated study information and registration of informed consent, but also randomization of participants, distribution of control interventions and screening surveys at baseline and follow-up. Aspects of RCTs that otherwise require extensive administrative effort, such as handling of informed consent, can be done automatically. In summary, the E-health study platform can be used to perform a research study and make sure that the same individual provides data at baseline and follow-up, even though the personal identity of this individual is unknown to the research team. This provides an ecologically valid way of evaluating interventions such as *myCompass*, which is not a health care intervention per se and where the idea is that the participant should be able to access the intervention without having to be enrolled as a patient in the health care system.

### Evaluation of myCompass

#### Participants

**Fig. 1:**
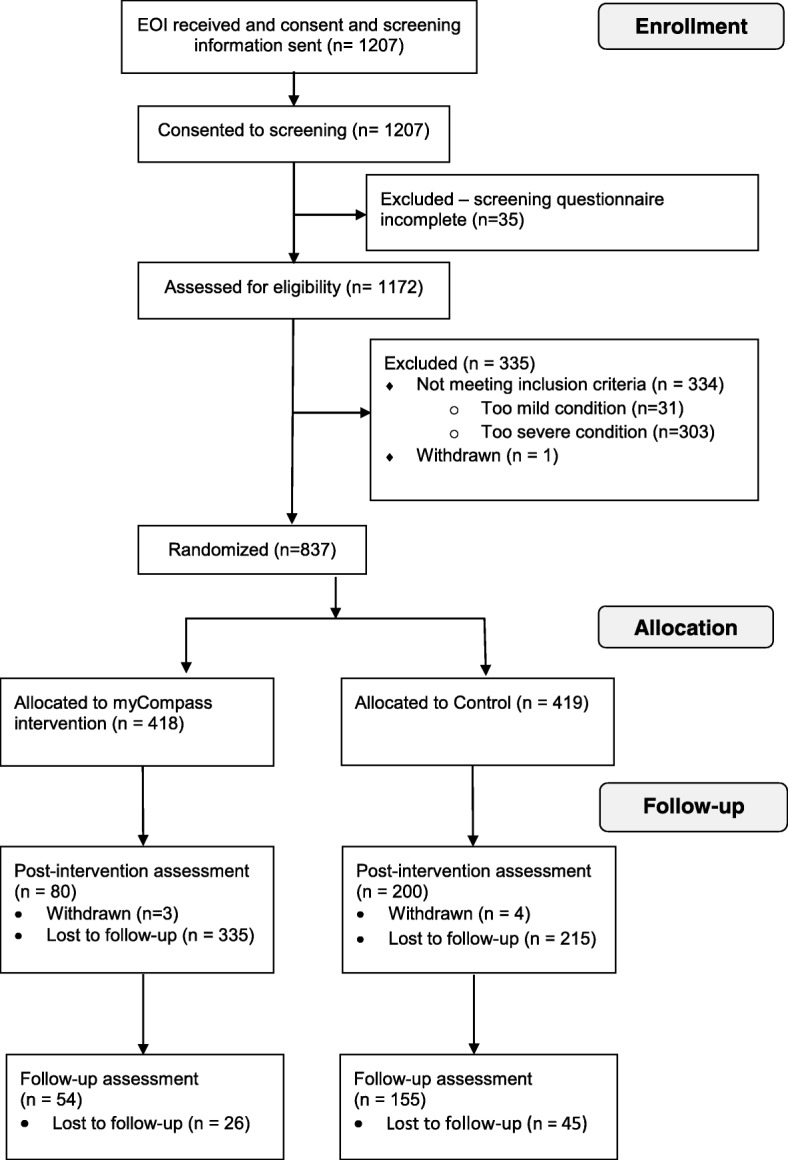
Flow-chart of participants. The flow-chart shows the flow of participants from initial enrollment to last follow-up survey

Participants were recruited through print advertisements (sent out to Karolinska Institutet and major Swedish newspapers), social media (e.g., Facebook, Twitter, blogs) and information in regular health care services. The study was also presented in live morning news in national TV (December 15, 2015). About half of participants signed up within a week of the appearance on TV, indicating that this was the most important recruitment tool. Individuals interested in participating signed up through the E-health study platform and completed the informed consent form. During the registration period (December 2015–January 2017), 1207 individuals enrolled and consented to screening. Out of those, 1172 provided complete screening surveys (i.e., the Patient Health Questionnaire-9 [PHQ-9) and the Generalized Anxiety Disorder 7-item scale [GAD-7); described below). Out of these, 837 were deemed eligible to participate and were randomized into *myCompass* (*n* = 418) and the control intervention (*n* = 419). For a complete overview of the participant flow, see Fig. 1.

Inclusion criteria were; Swedish resident 18 years or older; having a valid e-mail address and reporting symptoms of mild to moderate depression and/or anxiety (i.e., a PHQ-9 score between 5 and 20 and/or a GAD-7 score between 5 and 15). Individuals who reported severe depression (≥20 on PHQ-9), or anxiety (≥15 on GAD-7) and/or suicidal thoughts were excluded. They received a standardized e-mail explaining why they were not included, with a strong recommendation to contact their M.D. or the emergency services.

#### Procedure

Individuals who fulfilled the eligibility criteria were randomized to *myCompass* or the control condition in a fully automated process through the E-health study platform. Participants randomized to the intervention condition were directed to a page at the platform where they were prompted to take part in *myCompass* during the following weeks*.* Identification and participant consent was initially done through e-mail and personal identity number/BankID. The personal identity number was used to check that participants were 18 years old or older. Following this checkpoint, all personal data was deleted.

In June 2016 (six months post study launch), five questions were added to the baseline survey in order to gather descriptive information on the participants (gender, living situation, children < 18 years, education and employment status). Nine questions were also added to follow-up survey 1 to gather usability data. The main reason for adding the questions was to explore possible explanations for the high drop-out rates, that were becoming evident at this point. In total, 157 participants (intervention *n* = 78; control *n* = 79) responded to the extended base line survey and 72 participants (intervention *n* = 39; control *n* = 33) responded to the extended follow up 1-survey. The questions were added after the majority of participants finished the intervention, the results thus stem from a smaller sub-group of participants. Of those who completed the extended baseline survey, most participants were female (63 and 66% in the *myCompass group* and controls, respectively), were employed more than 50% (55 and 70% in the *myCompass* group and controls, respectively) and had post-secondary education (63 and 68% in the *myCompass group* and controls, respectively), see Appendix Table 5.

#### Baseline- and outcome measures

Participants completed two self-report measures online at baseline; post-intervention (seven weeks after inclusion), and at follow-up (19 weeks after the inclusion). Both measures were administered through the E-health study platform.

The **Patient Health Questionnaire-9** (PHQ-9; [35] is a 9-item measure that assesses degree of depressive symptoms. Items are scored between 0 (=*not at all*) to 3 (= *nearly every day*), with a total score of 27. A score between 0 and 4 indicates no depression; 5–9 minimal symptoms; 10–14 minor depression; 15–19 moderately severe major depression, and 20–27 severe major depression. PHQ-9 has demonstrated satisfactory psychometric properties with adequate internal consistency (Cronbach’s α ranging between .86–.89) and test-retest reliability (*r* = .84) [36].

The **Generalized Anxiety Disorder 7-item scale** (GAD-7; [37] is a seven-item measure that assesses levels of general anxiety disorder. GAD-7 is widely used as a general measure of anxiety, particularly in Swedish internet-based trials. Using GAD-7 makes comparisons to other, similar trials easier. Items are scored between 0 (= *not difficult at all*) to 3 (= *extremely difficult*), with a total score of 21. A score between 0 and 4 indicates no anxiety; 5–9 mild anxiety; 10–14 moderate anxiety; and 15–21 severe anxiety. GAD-7 has demonstrated satisfactory psychometric properties, with an internal consistency of α = .92, and test-retest reliability of *r* = .83 [36].

### Interventions

#### myCompass

The *myCompass* is a completely automated self-guided program without therapist contact. The program contains 12 different modules targeting common mental health problems (e.g. anxiety, sleep disturbances, depression and stress). The modules are based on principles from cognitive behavior therapy, interpersonal psychotherapy, problem solving therapy and positive psychology. The participant can choose what modules to work with or opt for a tailored program based on responses in an online self-monitor questionnaires completed at registration. Each questionnaire consists of several questions on a certain topic such as sleep deprivation or anxiety. Ratings are made on a 10-point scale, ranging from 0 (= *not at all*) to 10 (= *extremely*) on questions such as “how stressed have you been feeling”; “how depressed have you been feeling” (during the past few weeks). The system is dynamic by allowing participants to re-take the questionnaire at any time point and thus the recommended modules can change. The modules consist of text material, images and homework assignments on specific topics (e.g., “setting smart goals”, “solving problems”; “sleeping well”).

*myCompass* also contains self-monitoring functions allowing participants to record their symptoms and track changes over time. The program uses email reminders, quotes sent as text messages, other participants’ stories and links to further reading about mental health (for a detailed description of the Australian version of *myCompass, see* Proudfoot, Clarke [31], Harrison, Proudfoot [32]). In the current study, participants logged into *myCompass* through a web browser (on computer, tablet or mobile phone); no separate application for tablets/mobile phones were constructed.

#### Control condition

Participants randomized to the control condition received a standardized program of one email per week during seven weeks in total, encompassing information on depression, stress and anxiety. In contrast to the intervention, the control condition did not contain any information on specific strategies to handle ones’ mental health issues (i.e. there was no individualization of the content). The information used was based on the control condition in the Australian evaluation of *myCompass* but was complemented with information relevant to the Swedish context, such as references to Swedish organizations and health care services, as well as activities relevant to Sweden e.g. “surfing” was replaced by “skiing”. The information was intended to take approximately 10 min to read on a computer. Participants in the control group were offered the *myCompass* intervention when the control intervention was finished.

#### Statistical analyses

Statistical analyses were conducted using XLSTAT 2018.3 (Build 50,965). Repeated Measures ANOVAs were performed to calculate between-group differences across time among completers (i.e., participants who completed the survey at baseline and both follow-ups). Such per-protocol analyses are associated with the risk of overestimating results, and with many possible confounding variables, but interpreted with caution it could provide the opportunity to detect the maximum possible benefit of the intervention. Linear mixed models (LMM) were used to adhere to the principle of intention to treat, allowing us to analyze the results using the complete data set for all participants (i.e., also including those who had only provided baseline data), see for example Hesser [38] for a discussion on the use of LMMs in randomized experiments. Maximum likelihood was used to handle missing data, under the assumption that data was missing at random. Active users (i.e., those who completed more than five out of 19 self-monitoring questionnaires and commenced at least one module in *myCompass*) were analyzed separately with both ANOVAs and linear mixed models for a more profound understanding of the results.

## Results

Results are divided into: (1) adherence (also including number of self-monitor questions and modules completed in *myCompass*); (2) Repeated measures ANOVA’s for “completers”, and (3) mixed model outcomes for all included participants.

### Adherence

Table 1 summarizes adherence to the *myCompass* intervention (i.e., as indicated by number of log-ins; number of commenced and completed modules; number of completed self-monitoring questions and completed follow-up measures) in all users and the smaller subgroup of “active users”. It also contains data on completion on follow-up surveys in the *myCompass* and control group. The results demonstrate that active users (*n* = 35) logged into *myCompass* more often than the larger group of all users (average number of log-ins 29.9 and 5.7, respectively); commenced and completed a larger number of modules (2.7 versus .6 and 1.8 versus .2), and self-monitor questions (97.6 versus 20.0). Regarding completion of the first and second follow-up survey, figures were equal and markedly higher among *myCompass* active users and participants in the control group, compared with the larger group of *myCompass* users.

**Table 1:** Adherence to *myCompass*

	*myCompass* all users (*n* = 418)		*myCompass* active users^a^ (*n* = 35)		Control group (*n* = 419)
	Avg.	*SD*	Avg.	*SD*	
Avg. number of log-ins	5.7	15.8	29.9	38.2	–
Avg. number of modules commenced	0.6	1.0	2.7	1.7	–
Avg. number of modules completed	0.2	0.7	1.8	1.5	–
Number of *myCompass* self-monitor questions	20.0	61.0	97.6	158.8	–
Avg. number of days from registration to last log-in	15.6	43.5	84.5	96.0	–
Completed first follow-up survey	19%		49%		48%
Completed second follow-up survey	13%		40%		37%

*Note*. In the *myCompass* intervention, modules target mental health problems (e.g., anxiety, sleep disturbances, depression and stress)

^a^Active users are defined as participants filling out > 5 self-monitor questionnaires and commencing at least one module

**Fig. 2:**
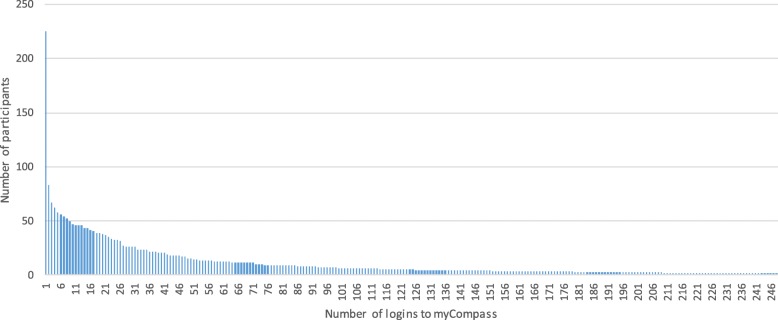
Number of log-ins to *myCompass*. Displays the number of participants who logged in to myCompass, and how frequently

Figure 2 illustrates total number of log-ins to *myCompass,* demonstrating an uneven pattern. In total, 41% of participants never logged in after registering and less than 50% of participants logged in ten times or more.

### Repeated measures ANOVA’s for completers

Table 2 demonstrates between-group differences across the three assessment points (i.e., at baseline and follow-ups) for “completers” in the two groups of *myCompass* users (all users and active users) and controls. Among controls, there were significant improvements on both outcome measures (i.e., PHQ-9 and GAD-7), with scores decreasing from baseline to second follow-up. There was no significant difference between groups, however.

**Table 2:** Repeated measures ANOVA for three subgroups, only including completers^a^

	Control group (*n* = 125)			*myCompass* all users (*N* = 28)			*myCompass* active users (*n* = 9)		
	Score	95% CI	*p*-value	Diff. from ctrl	95% CI	p-value	Diff from ctrl	95% CI	*p*-value
PHQ-9									
Pre-treatment	10.36	[9.58, 11.15]	–	0.71	[−1.12, 2.54]	0.447	1.42	[−1.61, 4.45]	.359
Post-t change	−3.11	[−3.86, − 2.37]	.000*	0.11	[− 1.36, 1.86]	0.900	0.11	[−2.77, 2.99]	.939
19 week change	−3.51	[−4.26, − 2.77]	.000*	−0.02	[−1.77, 1.73]	0.979	−1.93	[−4.81, − 0.95]	.188
GAD-7									
Pre-treatment	8.88	[8.24, 9.54]	–	0.08	[−1.45, 1.60]	0.922	−0.11	[−2.63, 2.41]	.932
Post-t change	−2.34	[−3.04, −1.65]	.000*	0.49	[−1.14, 2.11]	0.556	0.90	[−1.79, 3.58]	.511
19 week change	−2.36	[−3.06, −1.66]	.000*	1.00	[−0.62, 2.63]	0.226	0.58	[−2.10, −3.27]	.670

*Note. PHQ-9* The Patient Health Questionnaire-9. *GAD-7* The Generalized Anxiety Disorder 7-item scale

^a^Completers were defined as participants who completed all three surveys (at baseline and follow-ups)

^*^indicates a significant result at the 0.05 level

### Mixed model outcomes

Table 3 demonstrates results for mixed models of all participants in the *myCompass* group and the control group on both outcome measures. The results demonstrate a significant effect of time (i.e., scores on both measures decrease in both follow-up measures), however no significant effects of group. Table 4 also demonstrates results for mixed models of all participants in both subgroups of *myCompass* users (i.e., all users and active users) and the control group, on both outcome measures. Again, results demonstrate a significant effect of time, however no significant effects of group.

**Table 3:** Mixed models all users

(*N* = 837)	Score	95% CI	*p*-value	N
PHQ-9				
Pre-treatment	10.37	[9.96, 10.78]	–	–
Post-t change	−2.53	[−3.00, − 2.06]	.000*	–
19 week change	− 2.97	[− 3.50, − 2.44]	.000*	–
Control	0.00	–	–	419
*myCompass*	0.27	[−0.30, 0.83]	.353	418
GAD-7				
Pre-treatment	9.05	[8.73, 9.39]	–	–
Post-t change	−2.03	[−2.45, −1.62]	.000*	–
19 week change	−2.10	[−2.56, − 1.63]	.000*	–
Control	0.00	–	–	419
*myCompass*	0.24	[−0.20, 0.67]	.280	418

*Note. PHQ-9* The Patient Health Questionnaire-9. *GAD-7* The Generalized Anxiety Disorder 7-item scale

**Table 4:** Mixed models comparing controls, *myCompass* all users and *my Compass* active users

(*N* = 837)	Score	95% CI	*p*-value	N
PHQ-9				
Pre-treatment	10.37	[9.96, 10.78]	–	–
Post-t change	−2.54	[−3.01, − 2.07]	.000*	–
19 week change	−2.98	[− 3.51, − 2.45]	.000*	–
Control	0.00			419
*myCompass*	0.22	[−0.36, − 0.80]	.449	383
Active users	0.65	[−0.71, −2.01]	.350	35
GAD-7				
Pre-treatment	9.05	[8.73, 9.37]	–	–
Post-t change	−2.04	[−2.45, −1.62]	.000*	–
19 week change	−2.10	[−2.56, − 1.63]	.000*	–
Control	0.00	–	–	419
*myCompass*	0.24	[−0.21, 0.69]	.300	383
Active users	0.26	[−0.78, 1.30]	.625	35

Note. *PHQ-9* The Patient Health Questionnaire-9. *GAD-7* The Generalized Anxiety Disorder 7-item scale

## Discussion

This paper aimed to (*i*) share our experience of the E-health study platform (i.e., regarding security aspects, functionality) to which the *myCompass* intervention was linked, and (*ii*) report findings from the empirical evaluation of *myCompass* (i.e., prerequisites, execution, study outcomes), in a community sample. The E-health study platform proved to be an efficient tool to streamline study administration. The *myCompass* intervention rendered initial interest among a relatively high number of individuals, however failed to maintain engagement of those enrolled. A smaller subgroup of active users had a markedly higher activity, however their treatment results were not significantly better than those of controls.

### The E-health study platform

The E-health study platform enabled the *myCompass* study to be delivered with a minimum of involvement from the study team. Randomized controlled trials are normally costly and require a rather extensive administrative effort, even for rather small trials. Research studies are required to be operated in a rigorous manner, demanding researchers to pay great attention to details regarding participant integrity and data security. The E-health study platform enables randomization, informed consent and evaluation to be administered in a fully automated manner, which minimizes the risks for omissions related to the human factor. This set up is rather unique and could simplify investigations of various fully automated interventions to a low cost. As mentioned earlier, this could ideally facilitate the spread and evaluation of evidence based interventions to an almost indefinite number of patients.

Health care providers struggle to offer services targeting mental health problems that are effective, easily accessible and cost-efficient. The combined approach using the E-health study platform and interventions like *myCompass* could prove to be one valuable approach, given that it is completely automated and easily accessible to anyone with an internet connection. However, some words of caution regarding the E-health study platform should be noted. First, while human errors could threaten the validity of research studies, human contact could make participants more willing to participate, even if that contact is limited to data collection and the handling of informed consent. Second, the E-health study platform is separate from the *myCompass* platform, which required participants to log on to two separate platforms. This could have deterred some participants from remaining in the study. With time, the transitions between different platforms will likely be smoother, as the platforms improve technically.

### Empirical evaluation of myCompass

Despite the potential possibilities of self-guided mental health interventions [30], and the positive findings from the developer led evaluation of *myCompass* [31], the aim to transfer *myCompass* to a Swedish context and replicate the original empirical evaluation did not fully succeed. The study rendered substantial interest initially with 1207 individuals enrolling, but it generally failed to maintain the engagement of those who enrolled, especially in the intervention group. Few participants logged into *myCompass* more than once or twice, and 41% of enrolled participants never logged in. This indicates that many participants were undecided regarding the intervention and their own participation after they had enrolled, but before they had commenced the program. Participants in both groups significantly lowered their symptoms of depression and anxiety, but given the large number of dropouts, no firm conclusions can be made regarding the effectiveness of the program. With the caveat of the small study samples in mind, our findings do indicate that there was a lack of significant difference between *myCompass* and the active control. A smaller subgroup of active users (*n* = 35) had a markedly higher activity in the program, however their treatment results were not significantly better than those of the control group. It is worth noting that even among “active users”, there was a relatively modest use of *myCompass* modules overall (an average of 1.8 modules completed). Interestingly, the control condition completed a higher number of follow-up measures than the intervention group, possibly due to a ‘questionnaire fatigue’ experienced by the intervention group who were required to answer a number of questions also within the myCompass platform. For these reasons, it is impossible to draw any firm conclusions on the effectiveness of the intervention per se.

It is a known challenge that digital interventions without therapist contact have higher drop-out rates as well as small effect sizes [13, 26]. Also, transdiagnostic interventions may be more difficult to implement compared to diagnosis specific interventions, since they target a wider range of problems, and the participant thus may have to acquire more skills relative to programs focusing on a narrower problem area. While such factors may have affected the outcome of this study, the question remains why there were such marked differences compared to the original Australian study. While the mean number of completed modules in the Australian version was 1.6, it was 0.2 in Sweden, the participants logged in on average 14.7 times in Australia vs. 5.7 times in Sweden, attrition rate 56.7% in Australia vs. 87% in Sweden, and while the outcome measures were different, results in Australia were clearly superior to those in Sweden.

Information about the program was widespread, and given the number of responses on the website, the public was aware of its existence. Those who self-assessed their symptoms did report mental health problems – sometimes more severe than was intended for this particular program – indicating that we did reach a relevant target group. It is likely that the implementation of the Swedish version of *myCompass* did not fully take into account issues of *transportability,* however. Transportability usually refers to the process of implementing efficacious interventions to usual-care settings [39], and how differences in settings, organizations and culture could effect the efficiency of an intervention. It is possible that the composition and design of the *myCompass* platform better suited an Australian audience, and some of the images used might have come across as foreign in a Swedish context. For example, some text messages to the Swedish participants contained inspirational quotes predominantly citing famous Australians. Moreover, the platform contained several links to Australian organizations, and a handful of words on the platform were not translated from English to Swedish. This could create a sense of alienation from the program, especially since there was no therapist to counter doubts raised by these types of flaws. It is likely that a more thorough processing of the *myCompass* content would have rendered greater adherence and better outcomes, especially since other self-guided interventions for similar problems have proven successful, e.g. the Wellbeing Course [40]. There is also a possibility that the user interface design of *myCompass* appeared somewhat outdated, given that it was designed approximately five years prior to the Swedish participants enrolled in the study. Also, the technical development has meanwhile gravitated towards mobile phone applications, rather than web-based solutions, which might have exacerbated any sense of *myCompass* being outdated. On a more speculatively note, Sweden has been rated as the second most digitally advanced country in the world, with Australia on eleventh place [41]. This likely increases the bar for what is considered to be acceptable in terms of technology and interface design for the participants. A somewhat outdated platform, with some issues regarding cultural aptitude, and a slightly more tech-savvy audience might thus together explain some of the results.

While the results from the Swedish *myCompass* trial are substantially poorer than the Australian counterpart, the level of adherence is similar to other self-guided interventions where it is a common finding that 90% of participants withdraw after two sessions [30]. This makes it difficult to draw empirically based conclusions on the effectiveness of these types of programs. Given the far-reaching dissemination of *myCompass,* it could perhaps be regarded as a tailored public health intervention for prevention of mental health symptoms (i.e., rather than providing treatment per se). Public health interventions are generally more difficult to evaluate, not least since they target a whole population rather than individuals, see for example Jackson et al. (2004) for a discussion on public health methodology. While the measurable effect of *myCompass* is small, it could still be one of several factors raising awareness and promoting mental health change. There is also a possibility that individuals who enroll in this type of intervention also seek regular mental health care or attend support groups; people may try out different alternatives before committing to one. For example, therapist-guided internet-interventions were, and are, offered through regular health care for several of the issues targeted in this study, e.g. insomnia [42] and depression [43]. The availability of similar self-guided mobile phone applications for promoting mental health like *Shimdi* [44], and English language applications like *Woebot* [45] should also be mentioned. It is likely that some participants moved on to other alternatives. This relative abundance of treatment alternatives is in line with the proposed apotecharies of digital care [24], but it could have the downside of pushing clients into a constant search for an envisioned perfect treatment option.

## Conclusion

Several valuable lessons can be drawn from the implementation and evaluation of *myCompass* in a Swedish context. The number of participants initially registering demonstrates that self-guided interventions could constitute viable alternatives for many people seeking treatment. The number of people who remain active in the intervention is much more limited than the number of individuals initially signing up, however. Another lesson is that these types of interventions need to be carefully transported to new contexts and languages to appear credible. Interventions must not necessarily be “home-grown”, but a thorough processing involving pilot trials is likely needed before large-scale studies are conducted. Finally, the E-health study was a highly useful tool, indicating that it has the potential to facilitate any intervention study of this type. A challenge for the future will be to stay in par with rapidly developing techniques to provide secure and efficient internet-based interventions for various patient groups.

## Appendix

**Table 5:** Descriptive information for the subgroup of participants registered post May 2016, divided into *MyCompass* and controls

	*My Compass* (*n* = 78)	Controls (*n* = 79)
Gender		
Female	49 (63%)	52 (66%)
Male	27 (35%)	23 (29%)
Other/blank	2 (3%)	4 (5%)
Employment^a^		
Employed more than 50%	43 (55%)	55 (70%)
Employed less than 50%	14 (18%)	10 (13%)
Retired	21 (27%)	14 (18%)
Education		
No formal education	7 (9%)	2 (3%)
Elementary school	5 (6%)	0 (0%)
Secondary school	10 (13%)	22 (28%)
Post-secondary education (e.g. university education)	49 (63%)	54 (68%)

Note. ^a^Employment includes any participation in the labor force such as working or studying

## Abbreviations

CBT: Cognitive behavioral therapy
CG: Control group
EOI: Expression of interest
GAD-7: Generalized Anxiety Disorder 7-item scale
ICBT: Internet-delivered cognitive behavioral therapy
LMM: Linear mixed models
PHQ-9: Patient Health Questionnaire–9
